# “Testing Can Be Done Anywhere”: A Qualitative Assessment of Targeted Community-Based Point-of-Care Early Infant Diagnosis of HIV in Lusaka, Zambia

**DOI:** 10.9745/GHSP-D-21-00723

**Published:** 2022-06-29

**Authors:** Tannia Tembo, Helen Dale, Nobutu Muttau, Megumi Itoh, Dhelia Williamson, Chanda Mwamba, Albert Manasyan, R. Suzanne Beard, Mackenzie Hurlston Cox, Michael E. Herce

**Affiliations:** a Centre for Infectious Disease Research in Zambia (CIDRZ), Lusaka, Zambia.; b U.S. Centers for Disease Control and Prevention, Atlanta, GA, USA.; c University of Alabama at Birmingham, Birmingham, AL, USA.; d University of North Carolina at Chapel Hill, Chapel Hill, NC, USA.

## Abstract

**Introduction:**

Delayed HIV diagnosis in HIV-exposed infants (HEIs) results in missed opportunities for early antiretroviral therapy (ART), causing significant morbidity and mortality. Early infant diagnosis (EID) depends on the availability of accessible and reliable testing services. We explored the acceptability, appropriateness, and feasibility of deploying a targeted community-based point-of-care (POC) EID testing model (i.e., “community POC model”) to reach high-risk mother-infant pairs (MIPs) in Lusaka, Zambia.

**Methods:**

We conducted in-depth interviews with a purposive sample of health care workers, study staff, and caregivers in high-risk MIPs at 6 health facilities included in a larger implementation research study evaluating the community POC model. We defined “high-risk MIPs” as mothers who did not receive antenatal testing or an attended delivery or infants who missed EID testing milestones. Interviews were audio-recorded, translated, and transcribed verbatim in English. Content and thematic analysis were done using NVivo 10 software.

**Results:**

Health care workers (n=20) and study staff (n=12) who implemented the community POC model noted that the portability and on-screen prompts of the POC platform made it mobile and easy to use, but maintenance and supply chain management were key to field operations. Respondents also felt that the community POC model reached more infants who had never had EID testing, allowing them to find infants with HIV infection and immediately initiate them on ART. Caregivers (n=22) found the community POC model acceptable, provided that privacy could be ensured because the service was convenient and delivered close to home.

**Conclusion:**

We demonstrate the acceptability, appropriateness, and feasibility of implementing the community POC model in Zambia, while identifying potential challenges related to client privacy and platform field operations. The community POC model may represent a promising strategy to further facilitate active HIV case finding and linkage to ART for children with undiagnosed HIV infection in the community.

## Abstract (precis)

Community-based point-of-care testing is an acceptable, appropriate, and feasible strategy for improving access to HIV diagnostic services for high-risk HIV-exposed infants.

## Teaser

**Key Findings:**

- Our novel community-based point-of-care (POC) early infant diagnosis (EID) testing model was introduced into the national prevention of mother-to-child transmission/EID program and leveraged infrastructure from contemporaneous index testing and back-to-care programs.
- The POC model was perceived as lowering some structural barriers to accessing EID testing and may help reach high-risk mother-infant pairs who may have challenges otherwise accessing HIV testing in traditional health care settings.
- Because the service is closer to home, privacy must be ensured to address concerns about disclosing HIV status to community members and neighbors.

**Key Implications:**

- Implementation of the community POC EID testing model by lay health care providers using the m-PIMA HIV-1/2 Detect platform may help overcome structural barriers to EID access, increase coverage of EID services for HIV-exposed infants at high risk of HIV acquisition, and reinforce other ongoing HIV case-finding activities in the community.
- Routine education about the community-based POC EID model at strategic service delivery points within health facilities and communities may help address questions and potential misconceptions about the model, as well as increase overall community knowledge about EID services, particularly those offered in the community.

## INTRODUCTION

In 2020, 150,000 children were estimated to have newly acquired HIV globally, almost 4 times the Joint United Nations Programme on HIV/AIDS target for ending mother-to-child transmission of HIV worldwide.^1^ Sub-Saharan Africa accounted for 89% of those estimated new infections.^2^ In Zambia, a high HIV burden country in southern sub-Saharan Africa, an estimated 8,300 children were reported to have become newly infected with HIV in 2020.^3^^,^^4^

Although the number of new pediatric infections continues to exceed global targets, delays in early infant diagnosis (EID) of HIV persist, with more than one-third of infants and young children globally having missed opportunities for early HIV diagnosis in 2020.^1^ These delays result in missed opportunities for timely antiretroviral therapy (ART) initiation and are a significant cause of HIV-related pediatric morbidity and mortality.^5^^,^^6^ Indeed, late provision of HIV care and treatment undermines the benefits of ART and compromises survival.^7^ Globally, 46% of the world's 1.7 million children living with HIV (CLHIV) were not on lifesaving treatment in 2020.^1^ In sub-Saharan Africa, 38%–49% of CLHIV may not have a diagnosis and are missing lifesaving ART, contributing to 86,000 AIDS-related deaths annually in children aged 0–9 years.^8^ In Zambia, where 19,000 children died from AIDS-related illnesses in 2019, only 71% of HIV-exposed infants (HEIs) had a virologic test done within the first 2 months of life and 79% of those found to have HIV infection initiated ART, suggesting ongoing gaps in pediatric case finding and treatment.^9^

The World Health Organization (WHO) advocates for the elimination of mother-to-child transmission of HIV through multilevel interventions, including quality antenatal care and prevention services that provide timely identification, treatment, and care for pregnant women with HIV or syphilis, their sexual partners, and their infants.^10^ For HEIs, the availability of point-of-care (POC) technologies have created new opportunities to improve EID. These opportunities may also increase EID testing coverage and subsequently reduce HIV-related deaths as HEIs are initiated on lifesaving ART early.^11^ However, many barriers to testing and care for HEIs remain in resource-limited settings.

At an individual level, barriers to EID testing include HIV-related stigma, late presentation or non-return of mother-infant pairs (MIPs) to health facilities for HIV services, inadequate support for mothers to remain engaged in care, and lack of male partner involvement or low social cohesion between the couple or within the child's family.^12^ At the health organization and health system levels, several barriers remain, including suboptimal systematic screening of all infants for HIV exposure^13^^,^^14^; the need for multiple encounters with the health system to receive EID testing; lost or missing EID test results, or delays in reporting results from centralized laboratories; limited availability of client-centered EID services at busy health facilities^15^^,^^16^; and stock-outs of essential EID reagents and sample collection consumables.

In most health facilities in Zambia, routine EID practice still requires that dried blood spot (DBS) samples be collected and transported to specialized laboratories for polymerase chain reaction (PCR) testing. This process must overcome multiple barriers to be timely and effective, including long shipping distances to centralized laboratories, poor road networks, logistical and courier problems, and inadequately trained staff. As a result, these barriers can adversely affect result turnaround time and cause delays in result reporting to clinicians and caregivers and ART initiation for infants with new HIV infection.^17^

In Zambia, HIV guidelines mandate that health care workers (HCWs) provide HIV testing services at different service delivery points in every facility to increase access to timely HIV diagnosis and treatment. Health care providers may collect blood to perform a nucleic acid test on HEIs using a POC machine at the point of service delivery. Identification and phone contact of HEIs who have not returned to the facility for initial or follow-up EID testing is done, with varying fidelity, by community health workers who screen patient records in under-5 clinics, nutrition, outpatient, inpatient, EPI/immunization, and maternity departments. Because a limited number of laboratories perform DBS PCR testing and there are competing demands on HCW time, many MIPs face substantial barriers to EID testing access, particularly those who may have disengaged from HIV care.

In response to challenges with routine EID services in Zambia, POC testing has been recently introduced into the national EID network, with Zambia's 2020 national consolidated HIV guidelines allowing health care providers to perform a nucleic acid test at the POC where available.^18^ WHO prequalified m-PIMA HIV-1/2 Detect (i.e., “m-PIMA”) from Abbott (Scarborough, ME, USA), formerly known as the Alere q, is a portable, battery-operated POC platform that offers qualitative POC nucleic acid amplification testing for the detection of HIV types 1 and 2, with results in just 52 minutes. Pragmatic trials and other implementation research studies have shown the m-PIMA to increase EID testing coverage in several African settings and subsequently, the proportion of HIV-infected infants who initiate treatment.^19^^,^^20^

In response to challenges with routine EID services in Zambia, POC testing has been recently introduced into the national EID network.

While recent studies have established the acceptability, feasibility, and effectiveness of POC EID testing in facility-based settings in Africa,^21^ such as maternity wards and prevention of mother-to-child transmission (PMTCT) and immunization clinics, there is a dearth of information about its use in community settings and other nontraditional or highly decentralized contexts. This is a critical evidence gap because POC testing deployed in community settings may hold promise as a strategy to decongest health facilities and improve access to EID testing and timely ART for infants experiencing barriers to accessing facility-based services, particularly during the time of COVID-19. Additionally, when integrated within existing HIV index testing and back-to-care platforms, community-based POC EID testing may improve EID coverage for infants at the highest risk of HIV infection.

In this article, we aim to address this evidence gap by qualitatively describing implementation outcomes of feasibility, acceptability, and appropriateness for a novel community-based POC EID testing model employing the m-PIMA platform (i.e., the “community POC model”). Here, we report findings from a qualitative evaluation of the community POC model that examined the perspectives of MIPs, health care providers, and study staff and was part of a larger implementation research study called “CDC Detect.” The main objective of CDC Detect was to determine whether the community POC model could improve EID testing uptake and HIV case finding to close the pediatric HIV treatment gap in Zambia.

## MATERIALS AND METHODS

### Main Study Design

The CDC Detect study was conducted at 6 public health facilities and their surrounding communities (i.e., catchment areas) in Lusaka, Zambia—collectively referred to as study sites—from October 2018 to September 2020. For the main study, we aimed to estimate the effect of implementing our novel community POC model in the homes and other preferred community locations of “high-risk” MIPs who had fallen out of the PMTCT cascade. In the parent study, we collected quantitative data on primary outcomes of EID testing uptake and EID positivity, as well as on secondary outcomes of ART uptake, time to ART, and 3-month survival and retention on ART for infants living with HIV. High-risk MIPs were identified according to criteria that could be easily ascertained by routine record review at the primary health facility level in Zambia (Box).

BOX **Criteria for High-Risk Mother-Infant Pairs**

- Mother attended ≥1 antenatal visits without a documented HIV test result (unknown HIV status); or
- Mother delivered at home and did not return to a health facility for postnatal care, and thus they and their child were not tested for HIV; or
- Mother documented as HIV infected but never initiated antiretroviral therapy (ART); or
- Mother disengaged from HIV care after ART initiation/did not return for antiretroviral refills (i.e., lost to follow-up or experienced treatment interruption during pregnancy or postpartum); or
- Mother/caregiver did not return to the facility with their infant for milestone early infant diagnosis testing per national guidelines at 6 weeks of age, 6 months of age, and/or 6 weeks after cessation of breastfeeding.

The study assessed the effect of implementing a novel community POC model in the homes and other preferred community locations of “high-risk” MIPs.

### Qualitative Guides and Theoretical Framework

We sought to assess implementation outcomes of acceptability, appropriateness, and feasibility^13^^,^^22^ according to an empirically supported framework, the Conceptual Model of Implementation Research.^23^ In brief, this model posits that implementation strategies involving multiple stakeholders operating at different levels, including at the individual, organizational, and health systems levels, are required to introduce an evidence-based intervention into routine practice.^23^ Following this framework, semistructured in-depth interviews (IDIs) were developed to examine these outcomes according to established definitions, as well as to qualitatively describe the implementation context, processes, and strategies associated with the community POC model at the individual, organizational, and health system levels.^24^ We defined acceptability as stakeholder perceptions that the community POC model using m-PIMA was agreeable, palatable, or satisfactory, and was assessed based on the stakeholder's knowledge of, or direct experience with, community-based POC EID testing. Appropriateness was considered as the perceived fit, relevance, or compatibility of the community POC model. Feasibility was the extent to which stakeholders had experiences with the community POC model using m-PIMA that suggested it had utility, was suitable for the program context, and/or actually fit with the intended purpose of decentralized and more accessible EID testing.^25^ All IDI guides were piloted to test their suitability and refine questions before field use.

### Community POC Model

The community POC model was implemented by a study team, supported by and embedded within the routine Ministry of Health (MOH) PMTCT/EID program. The study team consisted of a study coordinator, data coordinator, data associates, research assistants (RAs), and peer educators (PEs), all of whom were employed by the implementing organization, the Centre for Infectious Disease Research in Zambia. The study team worked closely with MOH nurse midwives from the maternal, newborn, and child health (MNCH) units at the 6 study sites. Study investigators trained nurse midwives at each site to perform confirmatory tests using the m-PIMA on infants who had tested positive on the first test in the community. In addition to supporting confirmatory POC EID testing for high-risk MIPs, the nurse midwives continued to perform their regular facility-based clinical duties, including offering routine DBS-based EID testing for MIPs that did not meet high-risk criteria.

Study staff reviewed MIP contact information and leveraged the routine MOH PMTCT/EID program to perform MIP tracing and community-based testing using the m-PIMA platform. Specifically, the PEs, who had previously worked as community health workers at their respective study sites, were tasked with collecting locator information on identified MIPs and physically locating their residences on enrollment days and during home visits, respectively. The RAs were responsible for reviewing antenatal, ART, maternity/delivery, EID, postnatal, and under-5 registers to identify MIPs that met high-risk criteria. After reviewing these records, RAs contacted the mother or other caregiver in the high-risk MIP log by phone or, failing that, by home visit to set up appointments for POC infant EID testing at home and/or other preferred community location of the caregiver's choice (e.g., community health post, friend or relative's home, nearest health center) and to re-engage MIPs in the PMTCT/EID cascade.

The RAs were trained in lay counseling, DBS collection, and POC testing at the beginning of the study. During implementation, they collected blood samples by heel or finger prick following MOH guidelines and performed the first POC test, and, if needed in case of machine error, a second test unless the caregiver preferred that their HEI be tested at the health facility. Test results were provided on the same day and, if positive, the RA and PE accompanied the MIP to the study site within their catchment area for confirmatory testing by repeat POC testing and DBS collection for confirmatory PCR testing, and ART initiation by a trained health worker.

### Study Setting and Selection of Participants

IDIs were conducted with a purposive sample of HCWs, MIPs/HEI caregivers, and study staff (i.e., RAs and PEs) from the 6 study sites. All IDIs were conducted between March and April 2020. Purposive sampling was used to select participants from among the limited number of available HCWs and study staff and for MIPs meeting various high-risk criteria based on study eligibility criteria.^26^^,^^27^ Since sample size in qualitative research is not set a priori, we continued recruitment until thematic saturation was reached.^28^ Based on prior research by the Centre for Infectious Disease Research in Zambia in this population, we estimated that conducting approximately 20 IDIs per participant type would be sufficient to reach saturation based on the data and the Conceptual Model of Implementation Research.

MIP caregivers were assessed for qualitative study eligibility at the time of enrollment into the parent study. They were approached to complete qualitative study consent and enrollment procedures and to schedule the IDI. To be eligible to participate in an IDI, caregivers had to have received an HIV diagnosis during a previous pregnancy with resultant exposure of their infant to HIV, had an antecedent child who received a DBS PCR test, and had an HEI enrolled in the parent study and tested for HIV using the community POC model. A random number generator in Microsoft Excel was used to order and randomly select the first 4 caregiver potential participants from each study site. RAs scheduled appointments for IDIs via telephone and verified the caregiver's eligibility to participate and their preferred location for the interview.

HCWs (i.e., nurse midwives and HIV lay counselors) from the study sites who had been trained by the study team in the use of the m-PIMA were recruited for IDIs by open invitation. To be eligible, HCWs had to have been working in the PMTCT programs at government health centers where the parent study was being implemented and had to have some hands-on experience using the m-PIMA during study implementation. Of the 36 HCWs invited, 20 had prior practical experience with the platform (considered as having done at least 4 tests on the platform), including having performed POC EID testing during community outreach activities, and were enrolled. Owing to the limited number of HCWs with sustained exposure to the m-PIMA, RAs and PEs were included as key informants to provide further insight about the implementation of the POC community model.

### Data Collection, Management, and Analysis

The study coordinator and RAs interviewed the study participants in English, Nyanja, or Bemba based on participant preference. Interviewers took notes while digitally recording the interviews, which lasted approximately 1 hour each. Independent transcribers used a one-step process to translate and transcribe verbatim in English using Microsoft Word (Microsoft, Redmond, WA, 2015). Transcripts were imported into NVivo 10 software (QSR International, 2012) for content and thematic analysis. Before analysis, a codebook with deductive codes was developed, which was supplemented with inductive codes as emerging themes were identified during the coding process.^29^ Data saturation was assessed during thematic analysis and determined to have occurred when no new codes emerged and information obtained during caregiver interviews became redundant.

### Ethical Considerations

The study protocol was approved by the institutional review boards at the U.S. Centers for Disease Control and Prevention (CDC no. 6877), University of Alabama at Birmingham (UAB IRB-170330004), University of North Carolina Chapel Hill (UNC 17-1798), University of Zambia Biomedical Research Ethics Committee (UNZA BREC 014-09-16), and the Zambia National Health Research Authority. All participants provided written informed consent.

## RESULTS

### Overview

We conducted 54 IDIs with 22 HEI caregivers, 20 HCWs, and 12 study staff. Of all the participants recruited in the study, 94% were women. Tables 1 and 2 list the characteristics of the respondents by study site. We organize our results here based on themes of acceptability, appropriateness, and feasibility, each of which represent implementation outcomes from our conceptual framework, the Conceptual Model of Implementation Research. Wherever possible, for each theme we highlight stakeholder perspectives relevant to the multilevel context of community POC model implementation.

**TABLE 1.** Descriptive Characteristics of Respondents in an Assessment of a Targeted Community-Based Point-of-Care Early Infant Diagnosis HIV Testing Model, Lusaka, Zambia

	Health Care Workers (N=20) No. (%)	Study Staff (N=12) No. (%)	Caregivers (N=22) No. (%)
Sex			
Male	1 (5)	2 (17)	0 (0)
Female	19 (95)	10 (83)	22 (100)
Age, years			
19–28	1 (5)	1 (8)	2 (9)
29–38	5 (25)	5 (42)	9 (41)
39–48	6 (30)	5 (42)	5 (23)
49–58	3 (15)	1 (8)	0 (0)
≥59	1 (5)	0 (0)	0 (0)
Missing	4 (20)	0 (0)	6 (27)
Education			
Primary	0 (0)	0 (0)	12 (55)
Secondary	0 (0)	4 (33)	8 (36)
Tertiary	20 (100)	8 (67)	0 (0)
None	0 (0)	0 (0)	2 (9)
HCW			
Nurse	6 (30)		
Nurse/midwife	13 (65)		
Lay counselor	1 (5)		
Other		12 (100)	
Marital status			
Single	7 (35)	3 (25)	0 (0)
Married	11 (55)	6 (50)	22 (100)
Widowed	2 (10)	1 (8)	0 (0)
Divorced	0 (0)	2 (17)	0 (0)
Other	0 (0)	0 (0)	0 (0)
Years of work experience			
1–10	14 (70)	8 (67)	
11–20	4 (20)	4 (33)	
>20	2 (10)	0 (0)	
HCW department			
Family planning/PAC	2 (10)		
MNCH/maternity	16 (80)		
OPD/pediatric ART	2 (10)		
Employment Status			
Employed	20 (100)	12 (100)	3 (14)
Self-employed	0 (0)	0 (0)	12 (55)
Not employed	0 (0)	0 (0)	7 (32)
Years living with HIV			
1–5			8 (36)
6–10			10 (45)
11–15			3 (14)
≥16			1 (5)

Abbreviations: ART, antiretroviral therapy; HCW, health care worker; MNCH, maternal, newborn, and child health; OPD, outpatient department; PAC, postabortion care.

**TABLE 2.** Distribution of Participants by Health Center in an Assessment of a Targeted Community-Based Point-of-Care Early Infant Diagnosis HIV Testing Model, Lusaka, Zambia

	Health Center						Achieved Sample Size
	1	2	3	4	5	6	
Caregivers	4	4	3	4	4	3	22
Health care workers							
Nurse-midwife	4	2	4	3	2	4	19
Lay counselor	0	1	0	0	0	0	1
Study staff							
Peer educator	1	1	1	1	1	1	6
Research assistant	1	1	1	1	1	1	6
Total	10	9	9	9	8	9	54

### Theme 1: Acceptability

#### EID Testing Preferences and Satisfaction

With routine EID testing services, caregivers expressed concern that long turnaround times could affect the accuracy of the test results.

*I can say before [i.e., during standard DBS testing] they may even have been making some mistakes because the time the test results took was just too long. Now with the machine, the testing is being done properly.* —Caregiver 3002, Health Center 3

Caregivers also felt that they had to spend a long time to travel to their preferred health facility and often had to wait long hours in queues before they could access care.

*… It takes 30–40 minutes to walk to the clinic … and there are many people [before you arrive]. A long time ago, I would go very early, place my card on the queue [to secure a place so that the card is among the first] and return home to do house chores. Now, these days, [with the community POC model] things have changed, [it] takes … 2 hours.* —Caregiver 1004, Health Center 1

Caregivers thought that routine testing not only caused undue anxiety and emotional distress from the long result wait times but could also lead to unnecessary repeat infant blood draws from misplaced samples or results.

*The waiting period is agonizing. It's really stressful because you don't know what to expect for months … We had to provide a sample 3 times because of continuously missing results … Thus, denying children the chance to start medication in a timely manner if they come out positive.* —Caregiver 6005, Health Center 6

*I have personally had that experience where I provide a sample for my child then the results get lost. They don't come back. So we have to provide another sample, now the anxiety that is attached to waiting for the unknown is just too much … . It's stressful for any mother.* —Caregiver 5003, Health Center 5

The community POC model was seen as lowering some of the structural barriers to EID testing access, including opportunity costs from lost work or the need to pay transport costs to visit the health facility. Caregivers and study staff also noted the benefits of not needing to forego their routine activities in exchange for taking their HEI to a health facility.

*The other difference was that the machine [m-PIMA] testing [with the community POC model] was right there close to me…I didn't have to travel long distances to have my child tested… I was able to save on a bit of transport money.* —Caregiver 5003, Health Center 5

*I think that some people have to travel long distances and [are more likely] to be the people whose children miss their tests. They find it hard to come to the [health] facility if they do not have transport money.* —Study staff 4102, Health Center 4

*I was a bit busy. I was like [the community POC model] is very good. Instead of me saving money waiting and spending money like [on] transport, [or] you finding transport for me to go to the clinic. I [said] I should just let them [staff] come; that's how they came. Yes, I welcomed them they were very good people.* —Caregiver 5101, Health Center 5

*In the community the mother would even relax, when you are done with everything, she would even start doing something else whilst the test is being run, but at the facility, I think they really want to go back home and do some other things.* —Study staff 4101, Health Center 4

The community POC model was seen as lowering some of the structural barriers to EID testing access.

Some caregivers were of the view that the community POC model was more convenient for caregivers who may prefer receiving EID services for their HEIs in the home.

*There are some people maybe who may find the comfort of their home to be much more comfortable than a health facility … so I think, if we had such a service [community POC model] being provided close to the people, when they need it, where they need it, I think it would be greatly welcomed.* —Caregiver 5003, Health Center 5

Despite most interviewees highlighting satisfaction with various aspects of the community model using the m-PIMA HIV-1/2 Detect, some caregivers and HCWs were apprehensive about the method of blood collection and how the blood samples from infants and young children were being used. In particular, the cartridge feature, with insertion of a blood sample directly into the platform, raised some concerns that might undermine client acceptability and required client health education to address potential misconceptions.

*Okay with the myths and misconceptions when we just started, there were rumors like say no, they are drawing blood, putting blood in the machine. They [caregivers] would say, “so where is this machine taking the blood?” … after that, you explain to them how the machine works and once you collect blood from the infant, you show them the drop of blood…which goes in the … [machine], in that tube and once they see the blood that there is only a little blood which goes there then, they are like comfortable with it*. —Study staff 1101, Health Center 1

### Theme 2: Appropriateness

#### EID Testing Setting Compatibility

In this study, POC EID testing was performed in the participant's home or, if the caregiver was not comfortable, in the study vehicle or at another mutually agreed upon space like a family member's home or a nearby community health post. Overall, 13 of the 22 (59%) caregivers interviewed preferred to have their HEIs tested in the community. Preferences for community or facility testing often related to individual-level factors such as history of HIV status disclosure to partners, family members, and neighbors in the community and health organization factors such as the privacy and anonymity (or lack thereof) available in facility testing spaces. One respondent who confirmed experiencing anxiety about being “exposed” to neighbors before having their HEI tested at home revealed that they had a “happy” experience in the community as the test was done discreetly.

*When they asked to come and test the baby from home, I started thinking “What are these people coming to do at my home? What will my neighbors think?” But after they came, they just came as ordinary people such that even my neighbor didn't notice. So, it really made me happy.* —Caregiver 5002, Health Center 5

RAs who conducted home visits to perform POC testing were of the view that some caregivers welcomed the idea of being visited at home to carry out a POC test on their infants even though some were also skeptical about being visited at home.

*Initially, the women would feel nice. They would welcome the idea of us going [to their homes]. But once you get there, I don't know what goes on in their mind … they think that maybe you are joking and become anxious. Some would welcome us, we would even enter their house and do the test … but some would become scared and say, no, I wish you could come home but there is no privacy at my home … you can't come into my home because there are other people …* —Study staff 2101, Health Center 2

Other study staff thought that continued stigma would deter caregivers from having their infants and young children tested at home.

*… Stigma is still going on in the sense that when we do community work [and get to a household], people [neighbors] really want to know why you have gone to a [particular] household [especially] that you just look different. So, people [neighbors] will be asking why you are there [at caregiver's home] and mothers [caregivers] would refuse to be tested in their homes.* —Study staff 1101, Health Center 1

Caregivers who preferred facility-based testing thought that home-based testing, and particularly testing in a shared space, could run the risk of breaching an individual's privacy and could increase the risk of involuntary disclosure of HIV status due to gossip in the community.

*At home, you will be surprised that I share the yard with a lot of people and they can start talking about me.* —Caregiver 6002, Health Center 6

Caregivers who preferred facility-based testing thought that home-based testing could risk breaching an individual's privacy, including involuntary disclosure of HIV status.

Study staff were of the view that caregivers were uncomfortable with being visited at home owing to the proximity of neighboring houses.

*… the houses are kind of close together. So, you have people passing by and you know when you are parked in a certain place for a long time, people start wondering what you are doing at their [neighbor's] house.* —Study staff 3101, Health Center 3

Other caregivers expressed concern about the perceived quality of testing procedures done in the community.

*… testing at home, no! That one, no … I think they wouldn't do a good job. They can leave some things, then they can be doing things in a hurry. Everything is found at the clinic so it's better everything is done at the clinic not at home. So, for me, I prefer at the clinic.* —Caregiver 3004, Health Center 3

#### Perceived Fit of the Community POC Model

Study staff stated that they were able to carry the m-PIMA platform during community home visits without raising suspicion from neighbors of caregivers about the purpose of the visit, suggesting the compatibility of conducting POC testing within the context of community outreach activities.

*… When you are going into the community, you never really know where you are going … sometimes, we did tests in the most awkward places but it turned out alright … Once a client [mother] agreed to have her baby tested from home but she received some visitors while we were there … we had done already drawn a sample and needed to run it [on the POC]. The mother was no longer willing to have the test done in her home and she asked us to leave … We had to process the sample in the car.* —Study staff 3102, Health Center 3

Study staff were also of the view that if the POC model were to be scaled up to increase the uptake of EID, some of the tasks that were restricted to professional staff only should be performed by community health workers, especially since they already provided EID services during community outreach.

*The platform can be used both at the facility and in the community … at growth monitoring points. It would be helpful to have the platform used not only by the nurses [at the health facilities], but also by [trained] counselors [community health workers] since they do most of the testing when they provide under-five services in the community.* —Study staff 2201, Health Center 2

Caregivers observed that there was a marked decrease in the result turnaround time with the community POC model, suggesting that this new model could address the long-recognized problem of long turnaround times for routine EID testing.

*… the difference that I have personally observed is that with being followed at home and being tested using this machine [m-PIMA], the results come out there and then. [Otherwise], you can stay for 3 to 4 months without collecting the results [with routine testing].* —Caregiver 1001, Health Center 1

### Theme 3: Feasibility

#### Suitability of the m-PIMA for Community Settings

Study staff reported ease of use of the m-PIMA diagnostic platform in community settings owing to its portability and availability of on-screen prompts.

*… it's not difficult to use [the m-PIMA], no. It's not difficult to load the cartridge and insert it, … Even carrying it is easy as well, yes.* —Study staff 6602, Health Center 6

*The machine is easy to use due to the prompts that guide you through.* —Study staff 3301, Health Center 3

Study staff reported ease of use of the m-PIMA diagnostic platform in community settings owing to its portability and availability of on-screen prompts.

HCWs reiterated that using the m-PIMA diagnostic platform can be challenging without adequate training and consistent use.

*… with adequate training and practice, use of the machine [m-PIMA] could be easy as long as you follow instructions … At first it was …, you know when you are just learning, sometimes it may seem hard but after some time I got used.* —HCW 4102, Health Center 4

Study staff noted that charging the battery sufficiently enabled them to run several tests in the community without having to connect the m-PIMA platform to a power source. This operational characteristic enabled the study staff to continue processing tests even when there were power outages in Lusaka, which were recurrent during study implementation.

*The battery is very strong. If you charge it enough, you can run about eight tests in the community, so the battery is strong. You don't even have to use electricity at the household that you visit. You can do the test anywhere, in the car, or at the community school or in the home, it's portable.* —Study staff 5502, Health Center 5

Study staff noted infrequent delays in providing test results to MIPs that occurred owing to the platform having errors while running a test in the community. They associated these errors with times when the platform was exposed to high ambient temperatures.

*Sometimes the [m-PIMA] machine would behave funny. From nowhere you find that the cartridge which is inside cannot even come out. Sometimes it just shuts down.* —Study staff 3301, Health Center 3

*Most commonly, especially when it was very hot, for some reason we had many errors. The platform would give errors and initially you would mostly get an error in the first 15 or first 23 minutes of the test running. But when it was so hot, you would actually have a test run all the way through the whole 52 minutes and then you get an error after 52 minutes, which was something else.* —Study staff 2202, Health Center 2

#### Actual Fit, Utility, and Practicality of the Community POC Model

Based on their experiences with the community POC model, HCWs felt that it could be successfully incorporated into existing community activities and used to replace or complement routine DBS testing.

*There are some activities done [in the community], for example, … there are follow-ups of HIV-positive mothers and people go for growth monitoring. It [the community POC model] is helping because some mothers don't come to the facility [but] if you follow them there [in the community], you will find them and test their babies.* —HCW 1203, Health Center 1

HCWs felt that the community POC model could be successfully incorporated into existing community activities and used to replace or complement routine DBS testing.

Caregivers, HCWs, and study staff compared the testing procedures, efficiency, and overall utility between the community POC model and routine care and thought the community POC model fit well within the EID program context. Study staff indicated that the community model changed the way HEIs access HIV care.

*This study [the community model] helped us to track babies who were lost to follow-up and offer options for testing. They [caregivers] didn't just come to the facility but were offered testing in the comfort of their homes or some place in the community.* —Study staff 3102, Health Center 3

*The Alere q or point-of-care has really helped us…We have those mothers that are at high risk, those that have never attended antenatal or that had at one point attended antenatal but upon knowing that they are HIV positive, have stopped coming here [health facility]. They only reappear maybe when they start coming for under-5 clinic … Since we are screening all the cards [and other records], we are coming across them.* —HCW 5105, Health Center 6

HCWs also noted the potential practicality of the community POC model given the investment of time and effort required to communicate an EID test result to or follow up with an MIP under the standard of care.

*So, in the old system we used to make calls sometimes you find the phone is not reachable or it's a wrong number … when we used to locate them we [would] test the baby but it [would] take a month for the results to come out. But for the platform, once you follow up … the mother [she] is normally encouraged to receive the result the same day.* —HCW 2105, Health Center 2

HCWs and study staff shared experiences about the possibilities afforded by the community POC model to increase coverage of EID testing services for high-risk infants, and the likelihood of identifying HIV-infected infants and referring them for treatment.

*We had a large number of infants…who had never had any EID testing … and not yet started on ART…[With] the Alere q machine [m-PIMA] … we tested 3 babies [at once and] they were all HIV positive and it was good that there and then we initiated all of them on ART and they are doing fine.* —Study staff 1102, Health Center 1

HCWs welcomed the use of the m-PIMA in the community to facilitate immediate linkage to ART for infants newly diagnosed with HIV and recommended that the community POC model using m-PIMA be scaled up in the health system.

*They [patients] have found help early compared to waiting for the DBS test results which can at times take 3 months. I just want to suggest that the platform should be rolled out to all facilities … With this Alere [m-PIMA] machine…if the babies are found positive, help is found right away*. —HCW 1201, Health Center 1

## DISCUSSION

Unlike other studies focused on facility-based implementation of POC EID testing, this qualitative study demonstrates the acceptability, appropriateness, and feasibility of implementing mobile POC EID testing as part of a targeted, community-based model in a high HIV burden setting. We describe high acceptance of the model, which was driven by quick result turnaround times and the lowering of structural barriers for high-risk MIPs. We note that study staff and HCWs perceived the utility of the community POC model for HIV case finding and early treatment initiation. We also detail the suitability of deploying the m-PIMA in a mobile fashion within an MOH EID program, even in challenging community settings, owing to ease of platform use, especially when proper training and platform maintenance are provided. Before contemplating model scale-up, additional implementation issues must be addressed and overcome, including those relating to operating temperature, task shifting testing procedures to community health workers, maintaining an uninterrupted supply of m-PIMA consumables, and ensuring client privacy when testing outside traditional health facility settings.

Despite recent reductions in HIV-related pediatric morbidity and mortality, many CLHIV in resource-limited settings still have missed opportunities for accessing EID testing and lifesaving ART as recommended by WHO.^22^^,^^30^ Community-based POC EID testing may help overcome barriers to EID testing and reach high-risk MIPs who may have challenges otherwise accessing HIV testing in traditional health care settings or may have fallen out of care or experienced treatment interruption.^31^^,^^32^ Our novel community POC model was introduced into the national PMTCT/EID program and leveraged infrastructure from contemporaneous index testing and back-to-care programs.^33^ These programs may function as natural platforms in which to embed the community POC model since the same community health workers who conduct index testing or community tracing can use similar methods to also identify high-risk MIPs in the community and offer them EID testing. While index testing and back-to-care services provided a platform for the community POC model, careful consideration should be given to additional investments required for scale-up.^34^ For this community model, implementation costs were associated with the purchase of the m-PIMA platforms, cartridges, clinical supplies, and insurance, as well as health worker and study staff training, community mobilization, transportation, and program monitoring. Insofar as our model targets the most high-risk MIPs, the cost per case identified could be less than an untargeted community-based approach, but this hypothesis requires further study.

A study conducted in Durban, South Africa, highlights the mobility of mothers during their pregnancy and how this may pose a potential problem with MIP tracing and follow-up. Approximately 60% of the HIV-infected mothers could not be reached when contacted to follow up on their infants' HIV testing 4–6 months after the birth of their child.^35^ In our parent study, mothers/caregivers did not show similar mobility following study enrollment, as approximated by our (unpublished) enrollment and retention rates; however, high MIP mobility, particularly in urban settings, may make the community POC model difficult to implement. To address mobility, it is important that PMTCT/EID programs maintain accurate and up-to-date locator information for the mother/caregiver and provide multilevel support to the MIP to ensure longitudinal engagement in the program.

Caregivers noted several aspects of the community POC model that pointed to its acceptability and appropriateness. They mentioned satisfaction with not having to spend money to travel to health facilities for testing, not having to queue in the health facility, and not having to worry about their infant's HIV status for as long while awaiting POC test results.^11^^,^^36^ These findings were echoed by a study from Eswatini that showed POC testing delivered in a health facility reduced many of the costs and concerns related to travel time for caregivers who lived far from health facility EID services.^37^ In our study, caregivers and HCWs alike both highlighted the compatibility and fit of the community POC model within the MOH EID program, which they perceived as offering faster EID testing, fewer barriers to testing access, and more timely availability of results and treatment for HIV-infected infants. However, some caregivers in our study raised concerns about the lack of privacy during community-based POC EID testing and reported anxiety about inadvertent disclosure of HIV status to neighbors or family members from testing in the community.

Caregivers noted several aspects of the community POC model that pointed to its acceptability and appropriateness.

To address these concerns, based on the study team's experience, any future efforts to replicate or scale up community-based POC EID testing should generally follow normative guidance for other community-based testing modalities, such as community index testing^38^; ensure that clients and providers contract for a preferred testing venue well in advance of a testing appointment, be it in the home, a school, a community health post, or other trusted community setting; schedule the testing appointment in a private space at their chosen venue on a day and time less likely to draw attention from others; avoid providers wearing any identifying uniforms associated with HIV or other stigmatizing condition; and offer clients pragmatic support (e.g., transportation) to present to their nearest health facility if that is their preferred testing venue for privacy or other concerns.

Although they were relatively infrequent, repeat blood draws for testing and extended time for a home visit were attributed to occasional technical challenges and malfunctioning of the m-PIMA platform during community testing. Technical challenges with the platform have been documented elsewhere in the region, including within health facilities. In a study done at health facilities in Eswatini, HCWs associated increased wait times with frequent POC EID machine errors and high demand for testing services.^37^ In our study, study staff and caregivers thought that community-based POC EID testing would reduce the need for retesting due to misplaced or lost samples in facilities, which they indicated was common with traditional DBS-based PCR testing. Concerns about retesting were not trivial, as they generally related to worries surrounding the need to collect additional blood, which were perceived by some participants to be harmful to the infant or young child. Caregiver views were mixed about the idea of blood sample collection done in the home, with some finding comfort in witnessing firsthand how their child's blood was processed for testing and others finding the practice better suited to health facilities. Caregivers who had prior experience with DBS testing generally wanted to have their HEIs receive POC testing at home based on the assurance that they would have immediate access to the test result. Several caregivers expressed a preference for community POC testing, despite the occasional need for immediate retesting, rather than face more frequent retesting at facilities due to lost or misplaced DBS samples and/or EID test results.

Study staff and caregivers thought that community-based POC EID testing would reduce the need for retesting due to misplaced or lost samples in facilities.

To ensure the feasibility of the community POC model, several implementation strategies should be considered.^23^^,^^39^ First, study staff and HCWs noted that training and practical experience with the platform under supervision built their confidence for testing independently. Results from a pilot of birth testing using a blend of POC and DBS EID testing in Kenya suggest that to realize the utility of POC platforms, clinical staff must be fully trained on POC diagnostic procedures.^40^ However, our study found that even with intensified HCW training, HCW enthusiasm was limited for conducting repeat confirmatory testing with the POC platform because it created an extra step in the testing process. Second, findings from a study in Malawi suggest that low testing volumes or positivity may make it less cost-efficient to use POC devices.^41^ As such, using a tailored strategy, such as our targeted model, which is focused on the most at-risk MIPs who have fallen out of the PMTCT cascade, may be required to achieve greater efficiency, particularly in PMTCT clinics with decreasing maternal-to-child transmission rates. Indeed, data from EID programs focused on the most at-risk infants presenting to facilities have noted reductions in the numbers needed to test to identify an HIV case with a similar targeted approach.^42^ Third, task sharing the community POC model with community health workers and lay counselors may be one implementation strategy to increase human resource capacity to offer the model, and it was recommended by participants in our study. Inadequate HCW staffing levels, high workload, and other competing tasks at the study sites make implementing the community POC model by professional HCWs less feasible. With a recent shift to lay counselors providing EID services, further task sharing could enable additional personnel to participate in the community POC model, thereby lessening the workload of traditional health workers and increasing the possibility that EID testing services can be extended to those who need them most. Finally, conducting community education involving the community at large and not restricted to key stakeholders, as was the case at the beginning of our main study, can help build demand for an innovation like the community POC model. Extrapolating from qualitative data on facility-based POC EID testing in Malawi suggests that caregivers' decisions about accepting POC EID testing in the community can be influenced by their knowledge about EID testing procedures in the community and POC EID testing more generally.^43^

Both HCWs and study staff indicated that the POC diagnostic platform was appropriate and suitable for use in community-based service delivery settings. Owing to the availability of on-screen prompts, it was noted to be usable by anyone who had received training on the platform. These participants felt that the size of the POC diagnostic platform and availability of a long-acting and chargeable external battery enabled portable use during community visits. Currently, national guidelines in Zambia support the use of POC EID diagnostic platforms by trained health workers at health facilities.^18^ In Zimbabwe, HCWs preferred the POC diagnostic platform to the standard of care as it used fewer resources and did not require extensive technical expertise to operate.^44^

### Limitations

We acknowledge several limitations with our study. First, to capture qualitative data on the community POC model from all implementers, we interviewed study staff as well as HCWs. For HCWs, we initially sought to enroll 36 HCWs trained on our approach; however, only 58% of HCW participants reported consistent m-PIMA platform use during implementation of the community POC model (i.e., having performed at least 4 tests themselves [range 2–8]). Therefore, some HCWs who participated in interviews may not have had intimate familiarity with operating the m-PIMA or engaging in the community POC model. To complement these data with the perspectives of other front-line implementers with intimate familiarity with the community POC model, we interviewed study RAs and PEs responsible for field implementation of the model. Interviewing these staff may have introduced social desirability bias and may have led these participants to accentuate the positive features of the community POC model in their responses. However, their extensive engagement in the implementation process put them in an excellent position to assess key implementation factors driving the community POC model. Moreover, data from their responses strongly triangulated with information from caregivers and HCW participants who also spoke to the acceptability, appropriateness, and feasibility of our model. Second, considering that our novel community POC model was implemented in selected health facilities and their surrounding catchment areas in an urban setting, the findings of this study may not be generalizable to rural parts of Zambia or other settings in sub-Saharan Africa. Further research would help to clarify the use of the community POC model in rural settings where EID testing approaches face additional implementation challenges.

## CONCLUSIONS

Caregivers, HCWs, and study staff described targeted community-based POC EID testing focused on high-risk MIPs as acceptable, appropriate, and feasible for the urban Zambian context. Notwithstanding a handful of operational challenges, a need to further address client privacy, and calls for task sharing with community health workers and lay health providers to optimize implementation, our community POC model holds promise for extending the reach of EID testing to the most vulnerable HEIs in Zambia and for improving pediatric HIV case finding, timely EID test result reporting, and early initiation of ART for CLHIV.

## Notes

**Peer Reviewed**

**First published online:** June 17, 2022.
